# The Role of Magnetic Resonance Imaging to Inform Clinical Decision-Making in Acute Spinal Cord Injury: A Systematic Review and Meta-Analysis

**DOI:** 10.3390/jcm10214948

**Published:** 2021-10-26

**Authors:** Arash Ghaffari-Rafi, Catherine Peterson, Jose E. Leon-Rojas, Nobuaki Tadokoro, Stefan F. Lange, Mayank Kaushal, Lindsay Tetreault, Michael G. Fehlings, Allan R. Martin

**Affiliations:** 1 Department of Neurological Surgery, University of California, Davis, CA 95817, USA; aghaffarirafi@ucdavis.edu (A.G.-R.); catpeterson@ucdavis.edu (C.P.); 2 John A. Burns School of Medicine, University of Hawai’i at Mānoa, Honolulu, HI 96813, USA; 3 NeurALL Research Group, Escuela de Medicina, Universidad Internacional del Ecuador, Quito 170411, Ecuador; joleonro@uide.edu.ec; 4 Queen Square Institute of Neurology, University College London, London WC1N 3BG, UK; 5 Department of Orthopaedic Surgery, Kochi University, Nankoku 783-0000, Japan; nobuaki.tadokoro@gmail.com; 6 Department of Neurosurgery, University Medical Centre Groningen, 9713 GZ Groningen, The Netherlands; stefanlange@hotmail.nl; 7 Department of Neurosurgery, Medical College of Wisconsin, Milwaukee, WI 53226, USA; mayankkaushal@yahoo.com; 8 Department of Neurosurgery, Toronto Western Hospital, University of Toronto, Toronto, ON M5T 2S8, Canada; lindsay.tetreault89@gmail.com (L.T.); michael.fehlings@uhn.ca (M.G.F.)

**Keywords:** spinal cord injury, SCI, spine trauma, magnetic resonance imaging, MRI

## Abstract

The clinical indications and added value of obtaining MRI in the acute phase of spinal cord injury (SCI) remain controversial. This review aims to critically evaluate evidence regarding the role of MRI to influence decision-making and outcomes in acute SCI. A systematic review and meta-analysis were performed according to PRISMA methodology to identify studies that address six key questions (KQs) regarding diagnostic accuracy, frequency of abnormal findings, frequency of altered decision-making, optimal timing, and differences in outcomes related to obtaining an MRI in acute SCI. A total of 32 studies were identified that addressed one or more KQs. MRI showed no adverse events in 156 patients (five studies) and frequently identified cord compression (70%, 12 studies), disc herniation (43%, 16 studies), ligamentous injury (39%, 13 studies), and epidural hematoma (10%, two studies), with good diagnostic accuracy (seven comparative studies) except for fracture detection. MRI findings often altered management, including timing of surgery (78%, three studies), decision to operate (36%, 15 studies), and surgical approach (29%, nine studies). MRI may also be useful to determine the need for instrumentation (100%, one study), which levels to decompress (100%, one study), and if reoperation is needed (34%, two studies). The available literature consistently concluded that MRI was useful prior to surgical treatment (13 studies) and after surgery to assess decompression (two studies), but utility before/after closed reduction of cervical dislocations was unclear (three studies). One study showed improved outcomes with an MRI-based protocol but had a high risk of bias. Heterogeneity was high for most findings (I^2^ > 0.75). MRI is safe and frequently identifies findings alter clinical management in acute SCI, although direct evidence of its impact on outcomes is lacking. MRI should be performed before and after surgery, when feasible, to facilitate improved clinical decision-making. However, further research is needed to determine its optimal timing, effect on outcomes, cost-effectiveness, and utility before and after closed reduction.

## 1. Introduction

Traumatic injury to the spine is common and can have devastating consequences when resulting in spinal cord injury (SCI). Acute SCI has an estimated incidence of 750 cases per million annually, often affecting younger individuals and resulting in a substantial impact upon families and society [1]. Evidence-based management of SCI is primarily focused on the acute period, including careful immobilization and transport, avoidance of hypotension and hypoxia, and early surgical decompression [2,3,4,5].

Imaging plays a critical role in the initial evaluation of spinal trauma, and computed tomography (CT) has largely supplanted radiography in modern clinical algorithms [6]. CT is widely available and can quickly screen trauma patients for numerous injuries (head, spine, thorax, and abdomen), but the visualization of the spinal soft tissues is poor, including the spinal cord, intervertebral discs, and ligaments. In contrast, magnetic resonance imaging (MRI) provides detailed views of these structures, allowing detection of spinal cord compression, acute disc herniation, ligamentous injury, and epidural hemorrhage. However, MRI has not been widely incorporated into trauma protocols due to concerns over safety, availability, inconvenience, cost, time required, and the argument that MRI findings rarely change clinical decision-making. Surprisingly, in spite of numerous manuscripts investigating MRI in spinal trauma and SCI, high-quality studies that compare clinical decision-making with and without MRI are lacking [6,7,8]. The American Association of Neurological Surgeons (AANS) and Congress of Neurological Surgeons (CNS) published guidelines for acute cervical spine trauma and SCI in 2002 and updated these in 2013, but offered limited recommendations regarding the use of MRI beyond its utility for cervical collar clearance—no recommendations on the use of MRI in adult patients with SCI were offered [6]. A systematic review performed by Bozzo et al. (2011) [8] took a broader approach in evaluating the clinical utility of MRI, by considering various indirect lines of evidence; based on low-quality evidence, the authors offered a weak recommendation that MRI be performed in all patients with SCI when feasible, to direct management [8]. More recently, a multi-disciplinary group sponsored by AOSpine, AANS/CNS, and Ontario Neurotrauma Foundation developed clinical practice guidelines (CPGs) on five controversial topics in SCI that included a similarly weak recommendation based on very weak evidence that MRI should be used when feasible, to guide clinical decision-making in SCI [9]. However, this CPG was primarily based on expert opinion, as the systematic review that formed its evidentiary basis found only one study that examined MRI for clinical decision-making, and it had a high risk of bias due to methodological issues [7,10]. Overall, the efforts to synthesize the evidence have not provided sufficient guidance on the routine use of MRI in acute SCI; as a result, clinical practice among spinal surgeons and other clinicians remains highly variable.

The overarching aim of this review was to determine if performing an MRI in the acute phase of SCI yields useful clinical information, leading to improvements in patient care and outcomes. However, in view of previous reviews that revealed the paucity of literature directly addressing this question, we aimed to perform a more inclusive review seeking indirect evidence that answers the key questions (KQs) listed in Table 1. Hence, our review aims to synthesize the available direct and indirect evidence regarding the utility of MRI, to guide decision-making in the acute phase of SCI.

## 2. Materials and Methods

The systematic review was designed in accordance with the Preferred Reporting Items for Systematic Reviews and Meta-Analyses (PRISMA) and the Cochrane Handbook of Systematic Reviews of Interventions [11,12,13].

Only studies with human subjects published in the English language were included, with the search confined to randomized controlled trials (RCTs), cohort studies, case series, and case-control studies. Reviews, opinion articles, case reports, and case series with less than ten patients were excluded. A summary of the study’s design in PICO format (population, intervention, comparison, outcome), including inclusion and exclusion criteria, is found in Table 2. Studies of interest were those that included adults (16 years or older) with SCI in the acute phase (within 7 days of injury). Relevant studies that also included a small proportion of pediatric patients (<20%) were allowed after consideration by the authors, but were marked with an asterisk (*) in all tables. Relevant studies were required to utilize MRI in the acute phase (within 7 days) for the purpose of clinical decision-making (Table 1). Investigations that only examined the role of MRI for prognostication were excluded. The outcomes of interest were selected a priori based on previous studies, and are specified as KQs 1–6 listed in Table 1.

For KQ1, studies were only included if they calculated the diagnostic accuracy of MRI in reference to a gold standard measure (e.g., intraoperative findings) for the detection of specific pathological entities (spinal cord compression, disc herniation, ligamentous injury, epidural hematoma, fracture, or a spinal cord lesion/edema/contusion in the context of SCI without radiologic abnormality [SCIWORA]). For KQ2, studies were included that simply reported the frequency of abnormal MRI findings among the entities included in KQ1. For KQ3, studies were included if they examined how often obtaining an MRI alters clinical decision-making in SCI, including if surgery is required, when to operate, surgical approach, the need for instrumentation, which levels to decompress, or the need for reoperation after surgery. Comparative studies were also included that evaluated differences in decision-making between groups that did and did not undergo MRI. For KQ4, studies were included that reported data on the optimal timing of MRI in acute SCI, including before or after closed reduction, before or after surgery, within a certain time period, or studies that compared differences in timing of MRI between groups. Regarding KQ5, studies that reported the frequency of adverse events when performing MRI in SCI were included. Finally, for KQ6, comparative studies were included that evaluated differences in outcomes (neurological, functional, health-related quality of life) between patients that received an MRI versus those that did not.

Medical subheadings (MeSH) and text words related to acute spinal cord injury and magnetic resonance imaging were utilized for the search strategy. Medline, Embase, and Cochrane Central Register for Controlled Trials (CENTRAL, Wiley interface) were searched. A first search was performed between 1 January 1980 to 30 April 2016. The project was subsequently postponed, and a second search from 1 January 2016 to 26 August 2020 was completed with some overlap in dates to ensure no relevant studies were missed. The starting year of 1980 for the search was based on the timing of the first clinical MRI manuscripts being published in the 1980s [14]. In relevant literature and reviews, references were manually searched for additional studies, while use of Embase ensured gray literature was also screened. Other than dates, no database search limitations were utilized. The Appendix A provides the search protocols, including keywords. Specific search strategies were developed under guidance of library/information scientists with expertise in systemic review searches. Search results were imported to EndNote (Clarivate Analytics, Philadelphia, PA, USA) for the first search and Covidence (Covidence A/S, Melbourne, Australia) for the second search, to reduce data entry errors and bias (i.e., deduplicating references). All investigation reports were assessed for inconsistencies (e.g., design description, outcome presentation, total patients analyzed).

Two authors independently screened all titles and abstracts based on the eligibility criteria. Two authors reviewed each manuscript in full-text for inclusion, to assess eligibility for final inclusion and data extraction. Any discordances between reviewers during the abstract screening, full-text screening, or data-extraction phases were resolved with discussion and review by a third author. In compliance with recommendations from the Cochrane Handbook for Systematic Reviews of Interventions, the following data were compiled into a Microsoft Excel spreadsheet: author, publication year, journal citation, setting, inclusion and exclusion criteria, study design, study population, KQs addressed, and outcomes.

Data were placed into tables stratified by the KQ, enabling qualitative assessment. For simplicity, studied populations were categorized as SCIWORA when CT or radiographs showed no evidence of traumatic injury; otherwise, they were labeled as SCI (i.e., including cases with fracture or malalignment). For quantitative outcome data that were similarly reported across studies and their populations, a meta-analysis was conducted to calculate pooled results. In these cases, a chi-squared test for heterogeneity was performed and the I^2^ statistic was calculated. Analysis was conducted using R v4.0.2 Statistical Software (R Foundation for Statistical Computing, Vienna, Austria) [15].

Two authors independently performed risk of bias assessment according to the National Institutes of Health (NIH) Quality Assessment Tool [16]. Studies appraised as good had minimally low risk of bias, studies appraised as fair had moderately low risk of bias, and those appraised as poor had high risk of bias.

## 3. Results

**Figure 1:**
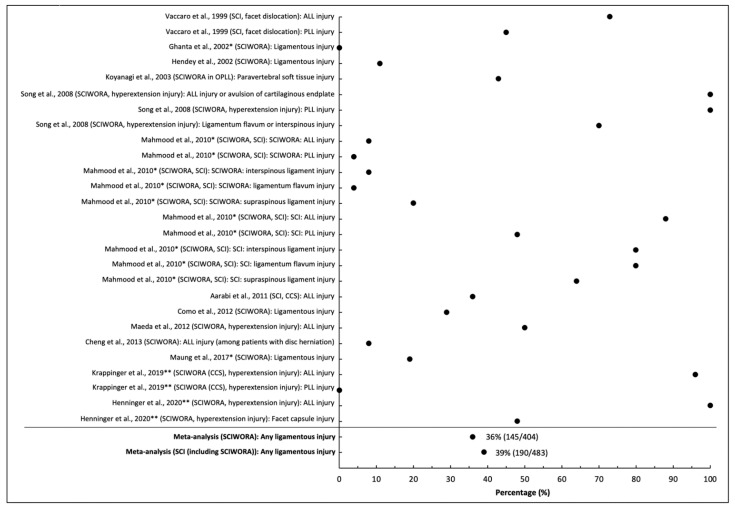
Frequency of ligamentous injury on MRI in patients with acute spinal cord injury. * Studies that include pediatric patients (<16) or unspecified age range. ** Studies with overlapping cohorts.

The two electronic database searches yielded a total of 21,323 unique citations (Figure 1). After title and abstract review, 268 manuscripts were selected. Following full-text review, 32 studies were identified that met eligibility criteria and were included in the qualitative synthesis in the form of Table 3, Table 4, Table 5, Table 6, Table 7 and Table 8. Three studies were prospective, while the remainder were retrospective case series, cohort studies, or case-control studies (Table 9). Risk of bias assessment found a high risk of bias in two studies, moderately low in 17, and minimally low in 13 (Table 9).

### 3.1. KQ1: Diagnostic Accuracy of MRI

Seven studies involving SCI were identified that addressed KQ1 (Table 3) [17,18,19,20,21,22,23]. Five studies calculated the diagnostic accuracy of MRI in relation to intraoperative findings [17,18,19,22,23], one compared against flexion/extension radiographs [20], and one against CT myelography [21]. Two studies with overlapping cohorts focused on hyperextension injuries and central cord syndrome [17,18]; both studies investigated detection of ALL injury, demonstrating superior sensitivity of STIR over T2-weighted (T2w) images (88% vs. 61%) [18] and a specialized MRI radiologist over a general radiologist (86% vs. 68%) [17]. In addition, one study also reported improved sensitivity of STIR over T2w images (82% vs. 61%) to identify intervertebral disc injury/herniation [18]. Two studies found 2 and 5 cases, respectively, of acute disc herniation on MRI that were verified by intraoperative findings [22,23]. Another study investigated the diagnostic accuracy of T2w images to detect intramedullary hemorrhage/contusion/edema in patients with SCIWORA compared with direct visualization of the spinal cord, reporting a sensitivity of 100% [19]. One study compared MRI against flexion/extension radiographs for segmental instability [20]; the MRI finding of ALL injury was present in 23/28 patients with instability (sensitivity: 0.82) and absent in 39/60 patients without instability (specificity: 0.65), while the finding of disc injury was present in 18/28 patients with instability (sensitivity: 0.64) and absent in 41/60 patients without instability (specificity: 0.68). One study found that MRI had only 40% (2/5) sensitivity to detect fracture, but 100% specificity (14/14) compared with CT myelography [21]. Meta-analysis was not possible for KQ1 findings due to the limited data available.

### 3.2. KQ2: Frequency of Abnormal Findings

Overall, 28 studies relevant to KQ2 were identified, reporting the frequency of certain pathological MRI findings in various types of SCI (Table 4) [17,18,19,20,21,23,24,25,26,27,28,29,30,31,32,33,34,35,36,37,38,39,40,41,42,43,44,45].

#### 3.2.1. Ligamentous Injury

Thirteen studies provided data on the frequency of ligamentous injury, all in patients with cervical SCI [17,18,20,24,25,26,27,28,29,34,37,41,43]. Among these studies, nine focused on patients with SCIWORA, in which the frequency of ligamentous injury ranged from 0 to 100% [20,24,25,26,27,28,34,37,41]. The pooled frequency of ligamentous injury in SCIWORA was 36% (145/404 across eight studies excluding [18] due to overlapping cohort with [17]), but heterogeneity across studies was high (I^2^ = 0.94, *p* < 0.001). Similarly, the pooled frequency of ligamentous injury was 39% in all patients with SCI (190/483 across 12 studies), with high heterogeneity (I^2^ = 0.93, *p* < 0.001; Figure 1).

#### 3.2.2. Disc Injury/Herniation

**Figure 2:**
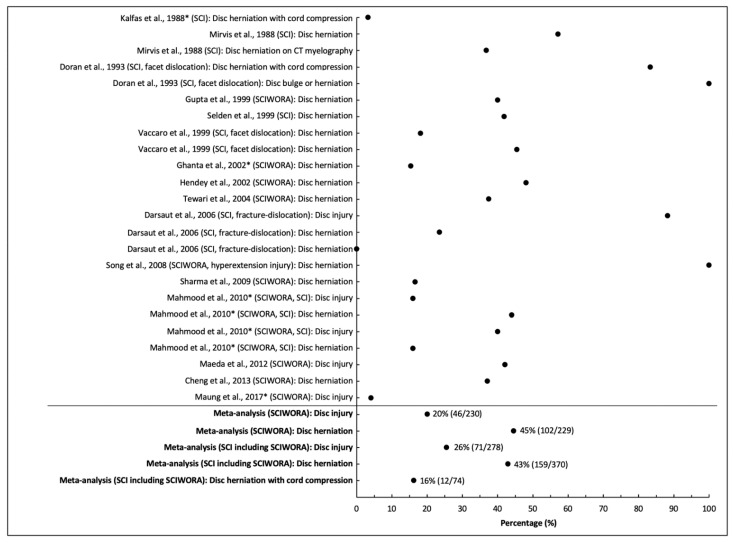
Frequency of disc injury or herniation on MRI in patients with acute spinal cord injury. * Studies that include pediatric patients (<16) or unspecified age range.

Sixteen studies provided data on the frequency of disc herniation and/or injury in SCI, including 15 in cervical injuries and one including all spinal levels [20,21,23,25,26,27,28,30,31,32,33,37,41,42,43,44]. The rate of disc injury ranged from 4% to 42% in studies involving cervical SCIWORA, whereas it was 40% to 88% in other SCI studies [20,21,23,25,26,27,28,30,31,32,33,37,41,42,43,44]. Disc herniation was present in 37% to 100% of patients with SCIWORA, whereas it was present in 24% to 100% in SCI [20,21,23,25,26,27,28,30,31,32,33,37,41,42,43,44]. Disc herniation causing cord compression varied from 3% to 83% in two studies involving SCI [23,30]. In SCIWORA, the aggregate rate of disc injury was 20% (46/230), while disc herniation was more frequent at 45% (102/229); both results showed high heterogeneity across studies (I^2^ = 0.96, 0.84, respectively, both *p* < 0.001). The pooled frequency of disc injury, disc herniation, and disc herniation causing cord compression across all studies (SCIWORA and SCI) was 26% (71/278), 43% (159/370), and 16% (12/74), respectively, while heterogeneity was high for all analyses (I^2^ = 0.95, 0.83, and 0.98, respectively, all *p* < 0.001; Figure 2).

#### 3.2.3. Cord Compression

**Figure 3:**
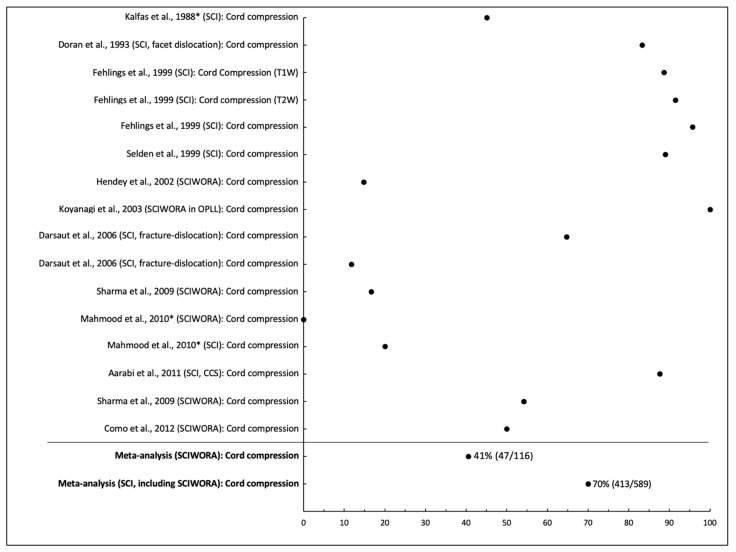
Frequency of cord compression on MRI in patients with acute spinal cord injury. * Studies that include pediatric patients (<16) or unspecified age range.

Twelve studies reported the frequency of ongoing spinal cord compression in SCI, including nine that included only cervical injuries, two that had sub-axial injuries (C3-T1), and one that included all levels [23,24,25,29,30,31,33,34,38,39,41,44]. A cohort examining sub-axial SCI found cord compression frequency at 89% (63/71) with a T1w sagittal sequence, but 92% (65/71) with T2w sagittal and 96% (68/71) when either result was positive [38]. In SCIWORA, cord compression was identified in 0% to 100% of patients in five studies [24,25,33,34,41]. In two studies involving cervical dislocations, cord compression was noted in 65% to 83% [30,31]. For fracture-dislocation patients, cord compression frequency was 65% (11/17) pre-traction, but 12% (2/17) post-traction according to one study [31]. The pooled frequency of cord compression across studies in patients with SCIWORA was 41% (47/116), whereas it was 70% (413/589) among all cases of SCI; heterogeneity across studies was high in both groups (I^2^ = 0.94, 0.95, respectively, both *p* < 0.001; Figure 3).

#### 3.2.4. Epidural Hematoma

**Figure 4:**
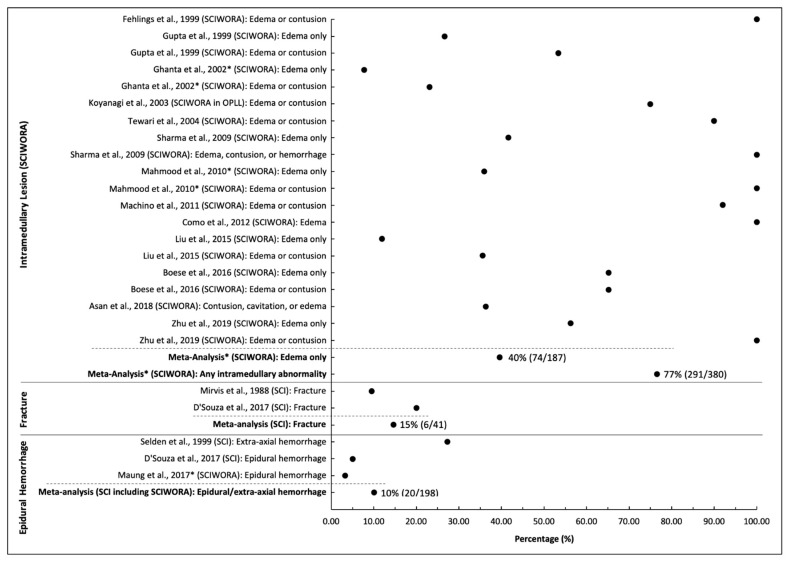
Frequency of Epidural Hemorrhage, Fracture, and Intramedullary Lesions (SCIWORA) on MRI in patients with acute spinal cord injury. * Studies that include pediatric patients (<16) or unspecified age range.

Three investigations in cervical SCI reported epidural hematoma in 3% to 27% of patients, resulting in a pooled frequency of 10% (20/198) [26,39,44]; the results showed high heterogeneity (I^2^ = 0.92, *p* < 0.001; Figure 4).

#### 3.2.5. Fracture

Two small studies provided data on the frequency of identifying fractures in patients with SCI, with a range of 10% to 20% and a pooled frequency of 15% (6/41) [21,39]; the results were homogeneous across these two studies, with I^2^ = 0, *p* = 0.61 (Figure 4).

#### 3.2.6. Intramedullary Lesions in SCIWORA

Thirteen studies provided data on the frequency of intramedullary signal change in patients with SCIWORA, including 336 cervical injuries and 44 thoracic injuries [19,24,32,33,34,35,36,37,38,40,41,42,45]. The pooled frequency of simple edema was 40% (74/187), while the rate of any intramedullary lesion (including edema, contusion, hemorrhage, or cavitation) was 77% (291/380) [19,24,32,33,34,35,36,37,38,40,41,42,45]; heterogeneity between studies was high (I^2^ = 0.90, 0.91 respectively, both *p* < 0.001; Figure 4).

### 3.3. KQ3: Influence of MRI on Clinical Decision-Making

Twenty studies provided data relevant to KQ3, regarding if surgery is required, surgical approach, when to operate, determining the need for instrumentation, which levels to decompress, and the need for reoperation after surgery, based upon MRI findings in acute SCI (Table 5) [10,17,21,22,23,24,26,28,29,30,31,32,33,37,40,42,44,45,46,47].

#### 3.3.1. If Surgery Is Required

Fifteen studies reported that MRI results directly influenced the decision of whether surgery was required in acute SCI [10,21,22,23,24,26,30,32,33,37,40,42,44,45,46]. Specific MRI findings that reportedly led to the decision for surgical treatment included cord compression, disc herniation, ligamentous injury, instability, and intramedullary edema (in conjunction with cord compression in SCIWORA). The frequency of MRI results reportedly leading to a decision to operate ranged from 3% to 100% across studies, with a pooled average of 36% (223/611) and high heterogeneity across studies (I^2^ = 0.96, *p* < 0.001).

#### 3.3.2. Surgical Approach

Nine studies reported on the influence of MRI findings on surgical approach [21,23,28,29,30,33,37,42,44]. Seven studies cited acute disc herniations with cord compression as the rationale for performing anterior surgery, at a rate of 3% to 83% of cases across studies [21,23,30,33,37,42,44]. Two additional studies of SCIWORA noted that MRI dictated surgical approach in all patients requiring surgery (42/211 and 70/70 patients, respectively), listing anterior compression, anterior compression limited to 1–3 segments, and kyphosis as reasons for selecting anterior surgery [28,29]. Overall, MRI was reported to affect the surgical approach in 29% (143/500) of patients in the included studies, with high heterogeneity (I^2^ = 0.97, *p* < 0.001).

#### 3.3.3. When to Operate

Three investigations examined the role of MRI in determining when to operate [10,31,44]. In two studies with overlapping datasets, 49% to 52% of patients required emergent surgery due to MRI-documented cord compression [10,44]. Two studies found that after traction/closed reduction, 33% to 82% of patients had good decompression and could undergo delayed surgery to perform definitive fixation [10,31]. Meta-analysis found that MRI affected surgical timing in 78% (65/83) of patients, with high heterogeneity (I^2^ = 0.84, *p* = 0.01).

#### 3.3.4. Need for Instrumentation

One study reported on the need for instrumented fusion due to the finding of segmental instability [17]. This study reported that the level of injury in SCIWORA (showing edema on MRI) and any levels showing ligamentous injury on MRI (19/23 patients) or segmental instability intraoperatively (22/23 patients) would be decompressed and fused.

#### 3.3.5. Which Levels to Decompress

A single study reported that MRI findings of edema and ligamentous injury, and intraoperative findings of instability, dictated which level(s) would be decompressed and fused [17].

#### 3.3.6. Need for Re-Operation after Surgery

Two studies reported the use of post-operative MRI to determine if adequate cord compression had been achieved after SCI [29,47]. One study found that 11/28 patients undergoing anterior surgery had residual cord compression, and this finding led to additional posterior surgical decompression [29]. Another study found that 63/184 patients had inadequate decompression following surgery for acute SCI, highlighting the role of cord swelling and the possible need for multi-level laminectomy and expansile duraplasty [47].

### 3.4. KQ4: When to Perform MRI

Sixteen studies provided data addressing KQ4 (Table 6) [10,22,23,24,26,28,29,30,31,32,33,43,44,45,46,47]. Fourteen studies concluded that MRI was useful during the initial assessment for the purpose of decision-making (related to one or more aspects of KQ3) [10,22,23,24,26,28,29,30,32,33,44,45,46,47]. However, two studies found that MRI prior to closed reduction of cervical facet dislocation was of unclear utility, with one study finding that two patients with pre-reduction disc herniation did not deteriorate after closed reduction [43], while another study similarly reported that 11 patients with pre-reduction cord compression did not deteriorate during closed reduction [31]. In contrast, Selden et al. found acute disc herniation in 10/18 patients with cervical dislocations, prompting a decision for immediate anterior surgery as the authors felt that closed reduction was unsafe [44]. Furthermore, Doran et al. reported neurological complications in three patients undergoing closed reduction of cervical dislocations that did not have pre-reduction MRI, and subsequent MRI showed disc herniations in all cases [30]. Three studies yielded data on MRI after closed-reduction, with two finding that it was helpful to identify ongoing spinal cord compression [31,44], whereas the third study found no neurological deterioration in spite of disc herniations in five of nine patients [43]. Two studies with overlapping cohorts reported that post-operative MRI was useful to identify inadequate decompression of the cord for consideration of re-operation [29,47]. No studies specifically recommended MRI within a set time period, but one study found no difference in the time interval from injury to pre-operative MRI between patients that were completely and incompletely decompressed [47].

### 3.5. KQ5: Frequency of Adverse Events When Performing MRI

Five investigations reported on frequency of adverse events when performing an MRI in patients with acute SCI (Table 7) [10,22,23,31,44]. Bao et al. examined patients receiving neutral, flexion, and extension MRIs for cervical SCI without fracture and dislocation, and amongst the cohort of 16 patients found no deterioration of neurological functions [22]. Similarly, when closed reduction for cervical dislocation was performed during MRI, no patients (*n* = 12) experienced permanent neurological deterioration or burning sensations at pin sites [31]. Pooled results found a 0% rate of adverse events (0/156 patients, 95% CI: 0% to 2.4%), with homogeneity across studies (I^2^ = 0, *p* = 1).

### 3.6. KQ6: Effect of MRI on Outcomes

One investigation addressed KQ6, evaluating differences in outcome between 66 patients assigned to an MRI-based treatment protocol (including urgent surgery) and 25 who were not assigned (due to a “contraindication to MRI, the need for an emergent surgical procedure, or the bias of specific admitting attending neurosurgeons regarding the ‘futility’ of emergent surgical treatment”) [10]. In patients assigned to the protocol group, Frankel grade improved from admission in 50%, relative to 24% in the non-protocol group (*p* < 0.006). Furthermore, eight of 50 patients from the protocol group presenting with complete motor quadriplegia (grade A or B) improved to independent ambulation (grade D or E), compared with none of the 20 reference patients (*p* = 0.09, Fisher exact test, not reported in original manuscript). MRI protocol patients also had shorter ICU stay (9.9 ± 1.7 days vs. 23.8 ± 3.7 days, *p* < 0.001) and total length of stay (71.4 ± 5.9 days vs. 99.9 ± 13.1 days, *p* = 0.02) [10]. Unfortunately, this study was deemed to have a high risk of bias due to non-random assignment to treatment groups and the confounding effect of more urgent spinal cord decompression in the protocol group compared with the reference group.

## 4. Discussion

This systematic review and meta-analysis addressed the role of MRI to inform clinical decision-making for patients with acute SCI, offering several lines of evidence supporting its use in routine practice. First, obtaining an MRI in the acute phase of SCI appears to be safe, with no adverse events reported in greater than 150 patients across five studies. This finding confirms the safety of obtaining an MRI in acute SCI, in spite of limited monitoring, additional transfers, and positioning the patient flat and supine for 30 to 45 min. MRI also demonstrates good diagnostic accuracy for ligamentous injury, instability, disc injury, disc herniation, and intramedullary tissue changes, albeit in a small number of comparative studies. Despite substantial heterogeneity between manuscripts, it is clear based on the large number of subjects and studies included in this meta-analysis that MRI frequently identified important pathological findings in patients with SCI, including spinal cord compression in 70%, disc herniation in 43%, ligamentous injury in 39%, and epidural hematoma in 10%. In patients with SCIWORA, MRI demonstrated intramedullary signal change in 77%, disc herniation in 45%, cord compression in 41%, and ligamentous injury in 36%. In contrast, evidence for the utility of MRI in detecting fractures in acute SCI was limited, with a low frequency of positive findings (15%) and poor diagnostic accuracy.

In terms of clinical decision-making, a large number of studies were identified that consistently reported evidence of clinical utility, influencing the decision to operate in 36% of patients, surgical approach in 29%, and the timing of surgery in 78%. Limited evidence also suggested that MRI is useful to determine the need for instrumentation, which levels to decompress, and if re-operation is needed for inadequate decompression. In terms of timing of MRI, most studies concluded that MRI should be performed on initial evaluation, prior to surgery. However, in cases of cervical dislocations, the utility of MRI prior to and after closed reduction remained unclear, due to conflicting results between studies regarding both the frequency and clinical significance of disc herniations; some reports suggest that MRI may be useful to avoid secondary injury due to a large disc herniation, but this area requires further study to draw conclusions. Finally, the results of this review confirm that evidence is lacking to directly show if obtaining an MRI improves outcomes; the only study addressing this topic had a high risk of bias due to non-randomized selection and a confounding effect of earlier spinal cord decompression in patients in the MRI-protocol group. Overall, the body of literature offers moderate evidence that (1) MRI is safe in the acute phase of SCI, (2) MRI has good diagnostic accuracy to detect certain features that are potentially useful for decision-making, (3) these features occur frequently, (4) these features often affect clinical decision-making, and (5) MRI should be performed prior to surgical treatment, whenever possible. However, further studies that investigate management decisions and clinical outcomes with and without MRI, the role of MRI in cervical dislocations, the time delay incurred by obtaining an MRI, and the cost-effectiveness of MRI are required to fully define its utility in acute SCI.

The novelty of the current study is that the review was broadly inclusive, looking for both direct and indirect evidence, while focusing narrowly on the topic of the role of MRI to facilitate clinical decision-making in patients with acute SCI. This review involved a comprehensive search of the literature that considered a large number of citations and full-text articles, and was designed to directly answer a common question that faces surgeons when a patient presents with acute SCI: should I get an MRI first, or just proceed directly to the operating room? The 2002 and 2013 AANS/CNS guidelines for the management of SCI also attempted to address the utility of MRI, but circumvented the main topic with only peripheral recommendations that MRI should be used in pediatric patients to assess SCI, or in adults for collar clearance, for the diagnosis of vertebral artery injury, or to assess patients with ankylosing spondylitis or SCIWORA [6]. Subsequently, AOSpine sponsored an effort to develop five guidelines for controversial topics in SCI, including one on the role of MRI in acute SCI, which provided a weak recommendation that MRI should be used in acute SCI to facilitate improved decision-making and prognostication [9]. These recommendations were based on a systematic review by Kurpad et al. (2017), which was unfortunately hampered by restrictive inclusion criteria yielding only one relevant study (which was deemed to have a high risk of bias) regarding the utility of MRI to guide acute SCI management, thus resulting in a vacuum of relevant evidence [7]. Conversely, Bozzo et al. (2011) utilized liberal inclusion criteria, involving the broader population of all spinal trauma, but was less focused and potentially lacked external validity. Furthermore, the review did not explore how individual studies reported changes in management based on MRI results, nor did it explore the importance of MRI in detection of spinal cord compression, which was the most common entity cited in the current review to affect management. In addition, the vast majority of studies included in both Bozzo et al. (2011) and Kurpad et al. (2017) investigated the use of MRI for prognostication, which we feel is of secondary importance, compared to the imperative task of deciding upon and planning surgical treatment. As a result, a knowledge gap currently exists regarding the optimal use of MRI, with highly variable practice patterns between surgeons. In summary, this systematic review provides a focused synthesis of the literature that clarifies the utility of MRI, while highlighting several areas that require further investigation.

SCI is an inherently difficult condition to study, due to profound heterogeneity in demographics, patterns of injury, timing after injury, neurological presentation, biomechanical stability, comorbidities, concomitant injuries, treatments performed, outcome measures, and MRI methods. The current study performed meta-analyses that clearly reflected this heterogeneity, providing aggregate results that may be helpful to provide general insights, but must be interpreted with caution as the frequency of findings varied with several factors. Complicating matters, the literature uses inconsistent definitions of terms such as SCIWORA, which was originally described in the pediatric population based on radiographs, but has increasingly been used to describe adult SCI without CT evidence of trauma (sometimes dubbed SCIWOCTET). Adult SCIWORA is widely felt to be considerably different than pediatric SCIWORA, with the former frequently involving degenerative spondylosis, ossification of posterior longitudinal ligament, and disc herniations, whereas the latter typically involves ligamentous laxity; therefore, it was not surprising that the SCIWORA results presented in this study also showed high heterogeneity. Furthermore, patients presenting with acute SCI frequently have concomitant injuries, hemodynamic instability, altered mental status, and/or undefined neurological deficits, making it difficult to develop recommendations that are universally applicable. However, the findings of this study are sufficiently compelling to suggest that MRI should be obtained during initial assessment of most patients with acute SCI, in the absence of a contraindication.

Looking ahead, further investigations should focus on several areas to elucidate the role of MRI in acute SCI. First, studies are needed that directly compare outcomes with and without MRI, while implementing similar management otherwise. However, it is doubtful that a randomized study can be ethically performed, as there was a perceived lack of equipoise expressed by expert clinicians in a recent guidelines effort [9]. Furthermore, emerging evidence suggests that earlier decompression has an hour-by-hour benefit on outcomes for the first 36 h after injury [48], suggesting that delays incurred in obtaining an MRI may counteract the benefits. Thus, future studies should include an analysis of the timing of surgery and the related impact of obtaining an MRI. On this topic, institutional protocols such as a “Code SCI” that streamline the care of SCI patients to minimize delays in imaging and definitive treatment should be developed [10,49], akin to “Code Stroke” protocols that have transformed stroke care. Future research is required that prospectively investigates the utility of MRI to make specific decisions on the need for surgery, surgical approach, the number of levels of decompression, and the need for instrumentation. Aarabi et al. (2019) demonstrated that decompressing more levels (up to five) with laminectomy showed higher rates of complete spinal cord decompression, likely due to greater alleviation of spinal cord swelling and secondary injury [47]; identifying pre-operative MRI features that predict spinal cord swelling could inform the need for additional levels of decompression and/or expansile duraplasty. In addition, the vast majority of previous studies have focused on cervical SCI, while the utility of MRI in thoracolumbar injuries is poorly defined, such as burst fractures with SCI. Evaluation of the cost-effectiveness of MRI is also needed to justify its widespread use, particularly in health systems and regions with scarce resources. Finally, emerging microstructural MRI techniques that measure specific physical properties such as axonal injury, demyelination, and perfusion should be studied for their potential value in prognostication [50].

This study is subject to several limitations. The systematic review involved two separate literature searches that were conducted using different interfaces and software tools, which occurred because the authors paused the initial project; this could have resulted in missed citations, although the literature search was comprehensive and overlapping dates were used to mitigate this risk. The large number of citations and full-text articles that were reviewed could also lead to errors, but we had multiple authors reviewing at each step to avoid errors. After careful consideration, we also modified the original inclusion criteria to allow studies with a small number of pediatric patients, as we felt that exclusion of certain key studies would result in a failure to identify important evidence; however, this decision potentially degrades the internal validity, as the small number of pediatric patients could mildly influence the overall results. There also exists the possibility of publication bias, which may have influenced our results. Our approach also excluded studies of spinal trauma without SCI or those that did not perform subgroup analyses with and without SCI; this approach omitted a large number of studies that offered substantial data on the diagnostic accuracy of MRI to detect ligamentous injury and fractures; however, we felt that it was essential to focus the current study on the specific clinical population of acute SCI.

## 5. Conclusions

MRI is safe and frequently identifies important findings with good diagnostic accuracy that alter clinical management in patients with acute SCI of all presentations, and thus, should be utilized when feasible. Therefore, pessimism that some surgeons feel toward obtaining MRI for the purpose of informing decision-making in acute SCI appears to be unjustified. Although the evidence is imperfect and indirect, it confirms the prior CPG recommendation “that MRI be performed in adult patients with acute SCI prior to surgical intervention, when feasible, to facilitate improved clinical decision-making”. Future prospective studies are needed to fully define the utility and cost-effectiveness of MRI in specific types of SCI, to allow for stronger recommendations that improve and standardize clinical practice.

## Figures and Tables

**Table 1:** Key Questions of Systematic Review.

Key Questions (KQ)
KQ1: What is the diagnostic accuracy of MRI to detect the following features that are likely to alter clinical management in patients with acute SCI?
1.1 Ongoing spinal cord compression 1.2 Disc herniation 1.3 Ligamentous injury 1.4 Epidural hematoma 1.5 Fracture 1.6 SCIWORA
KQ2: What is the frequency of abnormal MRI findings (from KQ1) in patients with acute SCI?
KQ3: How often does obtaining an MRI alter clinical decision-making in acute SCI??
3.1 If surgery is required 3.2 When to operate 3.3 Surgical approach (e.g., anterior vs. posterior) 3.4 Need for instrumentation 3.5 Which levels to decompress 3.6 Need for reoperation after surgery
KQ4: When should MRI be performed in acute SCI?
4.1 Before closed reduction 4.2 Before surgery 4.3 After closed reduction/surgery to assess decompression 4.4 Within a specific time period (e.g., 24 h)
KQ5: What is the frequency of adverse events when performing MRI in acute SCI patients?
KQ6: How does obtaining an MRI (compared with not obtaining MRI) affect neurological, functional, and health-related quality of life outcomes?

**Table 2:** PICO Summary of Inclusion and Exclusion Criteria.

	Inclusion	Exclusion
**Patient**		
	Adult human population (≥16 years old) Studies that include patients in the acute phase of SCI (within 7 days of injury)	Pediatric population (age < 16)
**Intervention**		
	MRI scan within 7 days of injury to inform one or more clinical decisions	MRI purely for prognosis
**Outcome**		
	Addresses one or more key questions in Table 1	
**Comparison**		
	MRI vs. no MRI MRI vs. CT No comparison (MRI alone)	
**Study Design**		
	Studies designed to assess the detection of a specific imaging feature and/or its relationship to alter decision-making or outcomes	Review articles Opinions Case reports or series < 10 patients Animal or biomechanical studies

**Table 3:** Key Question 1: What is the diagnostic accuracy of MRI to detect specific features of spinal injury that are likely to alter clinical management in patients with acute SCI.

Citation	Disease	Sample Size	Age (Years)	SCI Level	Sequence	Field Strength	Injury	Comparison		Outcome
**Ligamentous Injury**										
Maeda et al., 2012	SCIWORA, hyperextension injury	*n* = 88	Mean: 64, range: 33–89	Cervical	NS	NS	ALL injury	Instability on flexion/extension radiographs		Unstable: 23/28 (sensitivity: 0.82) Stable: 39/60 (specificity: 0.65)
Krappinger et al., 2019 **	SCIWORA, CCS, hyperextension injury	*n* = 23	Mean: 62.7, range: 38–87	Cervical	T1W, T2W, STIR	1.5T	ALL injury PLL injury	Intraoperative findings Intraoperative findings	Radiologist On-Call Specialized MRI Radiologist	Injured: 15/22 patients (sensitivity: 0.68), 15/25 segments (sensitivity: 0.60) Uninjured: 0, denominator NS (specificity: 1.0) Injured: 19/22 patients (sensitivity: 0.86), 22/25 segments (sensitivity: 0.88) Uninjured: 0, denominator NS (specificity: 1.0) 100% agreement, no injury in 23/23 patients
Henninger et al., 2020 **	SCIWORA, hyperextension injury	*n* = 21	Mean: 62, range: 38–87	Cervical	T1W, STIR	1.5T	ALL injury PLL injury	Intraoperative findings Intraoperative findings	STIR T2 Any sequence Any sequence	88% agreement 61% agreement 88% agreement PLL injured: 1/2 (sensitivity: 0.50)
**Fracture**										
Mirvis et al., 1988 *	SCI	*n* = 21	Mean: 42.5, range: 17–66	Cervical	T1W, T2W	1.5T	Fracture	CT myelography		Fracture: 2/5 (sensitivity: 0.40) No fracture: 14/14 (specificity: 1.0)
**Disc Injury/Herniation**										
Kalfas et al., 1988 *	SCI	*n* = 62	NS	Cervical (*n* = 40), Thoracic (*n* = 17), Lumbar (*n* = 5)	T1W, T2W	0.5T	Disc herniation with cord compression	Intraoperative findings		2/2 (sensitivity: 1.0)
Maeda et al., 2012	SCIWORA, hyperextension injury	*n* = 88	Mean: 64, range: 33–89	Cervical	NS	NS	Intervertebral disc injury	Instability on flexion/extension radiographs		Unstable: 18/28 (sensitivity: 0.64) Stable: 41/60 (specificity: 0.68)
Bao et al., 2020	SCIWORA	*n* = 16	Mean: 51.1, range: 30–73	Cervical	T1W, T2W	3.0T	Intervertebral disc injury	Intraoperative findings	T2W	5/5 (sensitivity: 1.0)
Henninger et al., 2020 **	SCIWORA, hyperextension injury	*n* = 21	Mean: 62, range: 38–87	Cervical	T1W, STIR	1.5T	Intervertebral disc injury	Intraoperative findings	STIR T2W Any sequence	88% agreement 61% agreement 79% agreement
**Cord Contusion/Edema**										
Zhu et al., 2019	SCIWORA	*n* = 16	Mean: 47.5, range: 22–65	Cervical	T2W	NS	Hemorrhage, contusion, or edema	Intraoperative findings	MRI	100% (16/16)

ALL, anterior longitudinal ligament; MRI, magnetic resonance imaging; CT, computed tomography; STIR, short T1 inversion recovery. * Studies that include pediatric patients (<16) or unspecified age range. ** Studies with overlapping cohorts.

**Table 4:** Key Question 2: What is the frequency of abnormal MRI findings of specific features of spinal injury that are likely to alter clinical management in patients with acute SCI?

Citation	Disease State	Sample Size	Age (Years)	SCI Level	Sequence	Field Strength	Injury	Outcome
**Ligamentous Injury**								
Vaccaro et al., 1999	SCI, facet dislocation	*n* = 11	Mean: 46, range: 17–84	Cervical	T1W, T2W	1.5T	ALL injury PLL injury	73% (8/11) 45% (5/11)
Ghanta et al., 2002 *	SCIWORA	*n* = 13 (subgroup)	Mean: 28.5, range: 0.4–78	Cervical	NS	NS	Ligamentous injury	0% (0/13)
Hendey et al., 2002	SCIWORA	*n* = 27	Median: 42, range: 21–89	Cervical	NS	NS	Ligamentous injury	11% (3/27)
Koyanagi et al., 2003	SCIWORA in OPLL	*n* = 28	Mean: 63.0, range: 45–78	Cervical	T2W	NS	Paravertebral soft tissue injury	43% (12/28)
Song et al., 2008	SCIWORA, hyperextension injury	*n* = 27	Mean: 54.1, range: 21–72	Cervical	T1W, T2W	1.5T	ALL injury or avulsion of cartilaginous endplate PLL injury Ligamentum flavum or interspinous injury	100% (27/27) 100% (27/27) 70% (19/27)
Mahmood et al., 2010 *	SCIWORA, SCI	SCIWORA: *n* = 25, SCI: *n* = 25	Mean: 45, range: 12–64	Cervical	T1W, T2W	0.5T	SCIWORA: ALL injury SCIWORA: PLL injury SCIWORA: interspinous ligament injury SCIWORA: ligamentum flavum injury SCIWORA: supraspinous ligament injury SCI: ALL injury SCI: PLL injury SCI: interspinous ligament injury SCI: ligamentum flavum injury SCI: supraspinous ligament injury	8% (2/25) 4% (1/25) 8% (2/25) 4% (1/25) 20% (5/25) 88% (22/25) 48% (12/25) 80% (20/25) 80% (20/25) 64% (16/25)
Aarabi et al., 2011	SCI, CCS	*n* = 42	Mean: 58.3, range: 32–87	Cervical	T2W, STIR	NS	ALL injury	36% (15/42)
Como et al., 2012	SCIWORA	*n* = 24	Mean: 60.5, range: 34–83	Cervical	T1W, T2W	1.5T	Ligamentous injury	29% (7/24)
Maeda et al., 2012	SCIWORA, hyperextension injury	*n* = 88	Mean: 64, range: 33–89	Cervical	NS	NS	ALL injury	50% (44/88)
Cheng et al., 2013	SCIWORA	*n* = 70	Mean: 57.7, range: 36–79	Cervical	T1W, T2W	NS	ALL injury (among patients with disc herniation)	8% (2/26)
Maung et al., 2017 *	SCIWORA	*n* = 123	NS	Cervical	NS	NS	Ligamentous injury	19% (23/123)
Krappinger et al., 2019 **	SCIWORA (CCS), hyperextension injury	*n* = 23	Mean: 62.7, range: 38–87	Cervical	T1W, T2W, STIR	1.5T	ALL injury PLL injury	96% (22/23) 0% (0/23)
Henninger et al., 2020 **	SCIWORA, hyperextension injury	*n* = 21	Mean: 62, range: 38–87	Cervical	T1W, STIR	1.5T	ALL injury Facet capsule injury	100% (21/21) 48% (10/21)
Meta-analysis ***	SCIWORA SCI (including SCIWORA)	*n* = 404 *n* = 482	Range: 0.4–89 Range: 0.4–89	Cervical Cervical			Any ligamentous injury Any ligamentous injury	36% (145/404), I^2^ = 0.94, *p* < 0.001 39% (190/483), I^2^ = 0.93, *p* < 0.001
**Disc Injury or Herniation**								
Kalfas et al., 1988 *	SCI	*n* = 62	NS	Cervical (*n* = 40), Thoracic (*n* = 17), Lumbar (*n* = 5)	T1W, T2W	0.5T	Disc herniation with cord compression	3% (2/62)
Mirvis et al., 1988	SCI	*n* = 21	Mean: 42.5, range: 17–66	Cervical	T1W, T2W	1.5T	Disc herniation	57% (12/21) 37% (7/19) on CT Myelography
Doran et al., 1993	SCI, facet dislocation	*n* = 12	Mean: 34.1, range: 18–59	Cervical	NS	NS	Disc herniation with cord compression Disc bulge or herniation	83% (10/12) 100% (12/12)
Gupta et al., 1999	SCIWORA	*n* = 15	Range: 20–60	Cervical	NS	NS	Disc herniation	40% (6/15)
Selden et al., 1999	SCI	*n* = 55	Mean: 29.2, range: 2–92	Cervical	T1W, T2W	1.5T	Disc herniation	42% (23/55)
Vaccaro et al., 1999	SCI, facet dislocation	*n* = 11	Mean: 46, range: 17–84	Cervical	T1W, T2W	1.5T	Disc herniation	Pre-Reduction:18% (2/11) Post-Reduction: 45% (5/11)
Ghanta et al., 2002 *	SCIWORA	*n* = 13 (subgroup)	Mean: 28.5, range: 0.4–78	Cervical	NS	NS	Disc herniation	15% (2/13)
Hendey et al., 2002	SCIWORA	*n* = 27	Median: 42, range: 21–89	Cervical	NS	NS	Disc herniation	48% (13/27)
Tewari et al., 2004	SCIWORA	*n* = 40	Mean: 42.1, range: 16–70	Cervical	T1W, T2W	NS	Disc herniation	38% (15/40)
Darsaut et al., 2006	SCI, fracture-dislocation	*n* = 17	Mean: 40.2, range: 19–78	Cervical	T1W, T2W	1.5T	Disc injury Disc herniation Disc herniation	Pre-Traction: 88% (15/17) Pre-Traction: 24% (4/17) Post-Traction: 0% (0/17)
Song et al., 2008	SCIWORA, hyperextension injury	*n* = 27 (subgroup)	Mean: 54.1, range: 21–72	Cervical (Lower)	T1W, T2W	1.5T	Disc herniation	100% (27/27)
Sharma et al., 2009	SCIWORA	*n* = 12	Mean: 38.66, range: 22–58	Cervical	T1W, T2W	NS	Disc herniation	17% (2/12)
Mahmood et al., 2010 *	SCIWORA, SCI	SCIWORA: *n* = 25, SCI: *n* = 25	Mean: 45, range: 12–64	Cervical	T1W, T2W	0.5T	SCIWORA: disc injury SCIWORA: disc herniation SCI: disc injury SCI: disc herniation	16% (4/25) 44% (11/25) 40% (10/25) 16% (4/25)
Maeda et al., 2012	SCIWORA	*n* = 88	Mean: 64, range: 33–89	Cervical	NS	NS	Disc injury	42% (37/88)
Cheng et al., 2013	SCIWORA	*n* = 70	Mean: 57.7, range: 36–79	Cervical	T1W, T2W	NS	Disc herniation	37% (26/70)
Maung et al., 2017 *	SCIWORA	*n* = 123	NS	Cervical	NS	NS	Disc injury	4% (5/123)
Meta-analysis	SCIWORA SCI (including SCIWORA)	*n* = 400 *n* = 577		Mixed			SCIWORA: disc injury SCIWORA: disc herniation SCI: disc injury SCI: disc herniation SCI: Disc herniation with cord compression	20% (46/230), I^2^ = 0.96, *p* < 0.001 45% (102/229), I^2^ = 0.84, *p* < 0.001 26% (71/278), I^2^ = 0.95, *p* < 0.001 43% (159/370), I^2^ = 0.83, *p* < 0.001 16% (12/74), I^2^ = 0.98, *p* < 0.001
**Cord Compression**								
Kalfas et al., 1988 *	SCI	*n* = 62	NS	Cervical (*n* = 40), Thoracic (*n* = 17), Lumbar (*n* = 5)	T1W, T2W	0.5T	Cord compression	45% (28/62)
Doran et al., 1993	SCI, facet dislocation	*n* = 12 (subgroup)	Mean: 34.1, range: 18–59	Cervical	NS	NS	Cord compression	83% (10/12)
Fehlings et al., 1999	SCI	*n* = 71	Mean: 39.7, range: 17–96	Sub-axial (C3-T1)	T1W, T2W	NS	Cord compression	T1W: 89% (63/71) T2W: 92% (65/71) Either: 96% (68/71)
Selden et al., 1999	SCI	*n* = 55	Mean: 29.2, range: 2–92	Cervical	T1W, T2W	1.5T	Cord compression	89% (49/55)
Hendey et al., 2002	SCIWORA	*n* = 27	Median: 42, range: 21–89	Cervical	NS	NS	Cord compression	15% (4/27)
Koyanagi et al., 2003	SCIWORA in OPLL	*n* = 28	Mean: 63.0, range: 45–78	Cervical	T2W	NS	Cord compression	100% (28/28)
Darsaut et al., 2006	SCI, fracture-dislocation	*n* = 17	Mean: 40.2, range: 19–78	Sub-axial (C3-T1)	T1W, T2W	1.5T	Cord compression	Pre-Traction: 65% (11/17) Post-Traction 6% (2/17)
Sharma et al., 2009	SCIWORA	*n* = 12	Mean: 38.66, range: 22–58	Cervical	T1W, T2W	NS	Cord compression	16% (2/12)
Mahmood et al., 2010 *	SCIWORA, SCI	SCIWORA: *n* = 25, SCI: *n* = 25	Mean: 45, range: 12–64	Cervical	T1W, T2W	0.5T	SCIWORA: cord compression SCI: cord compression	0% (0/25) 20% (5/25)
Aarabi et al., 2011	SCI, CCS	*n* = 211	Mean: 58.3, range: 32–87	Cervical	T2W, STIR	NS	Cord compression	88% (185/211)
Como et al., 2012	SCIWORA	*n* = 24	Mean: 60.5, range: 34–83	Cervical	T1W, T2W	1.5T	Cord compression	54% (13/24)
D’Souza et al., 2017	SCI	*n* = 20	Mean: 35.95, range: 17–54	Cervical	T1W, T2W	3T	Cord compression	50% (10/20)
Meta-analysis	SCIWORA SCI (including SCIWORA)	*n* = 116 *n* = 589	Range: 17–96	Mixed			Cord compression Cord compression	41% (47/116), I^2^ = 0.94, *p* < 0.001 70% (413/589), I^2^ = 0.95, *p* < 0.001
**Epidural Hematoma**								
Selden et al., 1999	SCI	*n* = 55	Mean: 29.2, range: 2–92	Cervical	T1W, T2W	1.5T	Extra-axial hemorrhage	27% (15/55)
D’Souza et al., 2017	SCI	*n* = 20	Mean: 35.95, range: 17–54	Cervical	T1W, T2W, DTI	3T	Epidural hemorrhage	5% (1/20)
Maung et al., 2017 *	SCIWORA	*n* = 123	NS	Cervical	NS	NS	Epidural hemorrhage	3% (4/123)
Meta-analysis	SCI (including SCIWORA)	*n* = 143	Range: 17–54	Cervical			Epidural/extra-axial hemorrhage	10% (20/198), I^2^ = 0.92, *p* < 0.001
**Fracture**								
Mirvis et al., 1988	SCI	*n* = 21	Mean: 42.5, range: 17–66	Cervical	T1W, T2W	1.5T	Fracture	10% (2/21)
D’Souza et al., 2017	SCI	*n* = 20	Mean: 35.95, range: 17–54	Cervical	T1W, T2W, DTI	3T	Fracture	20% (4/20)
Meta-analysis	SCI	*n* = 41	Range: 17–66	Cervical			Fracture	15% (6/41), I^2^ = 0, *p* = 0.61
**Intramedullary Lesion (SCIWORA)**								
Fehlings et al., 1999	SCIWORA	*n* = 14 (subgroup)	Mean: 39.7, range: 17–96	Sub-axial (C3-T1)	T2W	NS	Edema or contusion	100% (14/14)
Gupta et al., 1999	SCIWORA	*n* = 15	Range: 20–60	Cervical	NS	NS	Edema only Edema or contusion	27% (4/15) 53% (8/15)
Ghanta et al., 2002 *	SCIWORA	*n* = 13 (subgroup)	Mean: 28.5, range: 0.4–78	Cervical	NS	NS	Edema only Edema or contusion	8% (1/13) 23% (3/13)
Koyanagi et al., 2003	SCIWORA in OPLL	*n* = 28	Mean: 63.0, range: 45–78	Cervical	T2W	NS	Edema or contusion	75% (21/28)
Tewari et al., 2004	SCIWORA	*n* = 40	Mean: 42.1, range: 16–70	Cervical	T1W, T2W	NS	Edema or contusion	90% (36/40)
Sharma et al., 2009	SCIWORA	*n* = 12	Mean: 38.66, range: 22–58	Cervical	T1W, T2W	NS	Edema only Edema, contusion, or hemorrhage	42% (5/12) 100% (12/12)
Mahmood et al., 2010 *	SCIWORA	*n* = 25 (subgroup)	Mean: 45, range: 12–64	Cervical	T1W, T2W	0.5T	Edema only Edema or contusion	36% (9/25) 100% (25/25)
Machino et al., 2011	SCIWORA	*n* = 100	Mean: 55, range: 16–87	Cervical	T2W	1.5T	Edema or contusion	92% (92/100)
Como et al., 2012	SCIWORA	*n* = 24	Mean: 60.5, range: 34–83	Cervical	T1W, T2W	1.5T	Edema	100% (24/24)
Liu et al., 2015	SCIWORA	*n* = 59	Mean: 41.1, range: 21–68	Cervical (*n* = 19), Thoracic (*n* = 40)	NS	3T	Edema only Edema or contusion	12% (7/59) 36% (21/59)
Boese et al., 2016	SCIWORA	*n* = 23	Mean: 53.7, range: 22–80	Cervical	T1W, T2W	1.5T	Edema only Edema or contusion	65% (15/23) 65% (15/23)
Asan et al., 2018	SCIWORA	*n* = 11	Range: 28–81	Cervical (*n* = 7), Thoracic (*n* = 4)	NS	NS	Contusion, cavitation, or edema	36% (4/11)
Zhu et al., 2019	SCIWORA	*n* = 16	Mean: 47.5, range: 22–65	Cervical	T2W	NS	Edema only Edema or contusion	56% (9/16) 100% (16/16)
Meta-Analysis *	SCIWORA	*n* = 380	Range: 12–87	Cervical (*n* = 336), Thoracic (*n* = 44)			Edema only Any intramedullary abnormality	40% (74/187), I^2^ = 0.90, *p* < 0.001 77% (291/380), I^2^ = 0.91, *p* < 0.001

* Studies that include pediatric patients (<16) or unspecified age range. ** Studies with overlapping cohorts. *** Henninger et al., 2020 was excluded from meta-analysis due to overlapping cohort with Krappinger et al., 2019.

**Table 5:** Key Question 3: How often does obtaining an MRI alter clinical decision-making in acute SCI.

Citation	Disease State	Sample Size	Age (Years)	SCI Level	Sequence	Field Strength	MRI Finding and Change in Decision-Making	Outcome
**If Surgery Is Required**								
Kalfas et al., 1988	SCI	*n* = 62	NS	Cervical (*n* = 40), Thoracic (*n* = 17), Lumbar (*n* = 5)	T1W, T2W	0.5T	2 patients had cord compression due to acute disc herniation leading to anterior surgery	3% (2/62)
Mirvis et al., 1988	SCI	*n* = 21	Mean: 42.5, range: 17–66	Cervical	T1W, T2W	1.5T	3 patients with disc herniation were managed with anterior decompression	14% (3/21)
Doran et al., 1993	SCI	*n* = 12 (subgroup)	Mean: 34.1, range: 18–59	Cervical	NS	NS	10 patients with frank disc herniation and severe cord compression were managed with anterior cervical discectomy	83% (10/12)
Gupta et al., 1999	SCIWORA	*n* = 15	Range: 20–60	Cervical	NS	NS	6 patients had intervertebral disc prolapse, all underwent anterior surgery	40% (6/15)
Selden et al., 1999 **	SCI	*n* = 55	Mean: 29.2, range: 2–92	Cervical	T1W, T2W	1.5T	Among 18 patients with bilateral dislocated facets, acute disc herniation in 10/18 led to anterior surgery Among 26 patients who underwent successful closed reduction, ongoing cord compression in 13/26 led to surgery	56% (10/55) 50% (13/26)
Ghanta et al., 2002 *	SCIWORA	*n* = 13 (subgroup)	Mean: 28.5, range: 0.4–78	Cervical	NS	NS	1 patient with disc herniation was managed with anterior decompression	8% (1/13)
Papadopoulos et al., 2002 **	SCI	*n* = 66	Mean: 32, range: 2–92	Cervical	T1W, T2W	1.5T	34 patients had cord compression leading to emergent surgery	51% (34/66)
Tewari et al., 2004 **	SCIWORA	*n* = 40	Mean: 42.1, range: 16–70	Cervical	T1W, T2W	NS	3 patients with disc herniation were managed with anterior decompression	8% (3/40)
Sharma et al., 2009	SCIWORA	*n* = 12	Mean: 38.66, range: 22–58	Cervical	T1W, T2W	NS	2 patients had disc prolapse and underwent surgery due to this finding	17% (2/12)
Machino et al., 2011	SCIWORA	*n* = 100	Mean: 55, range: 16–87	Cervical	T2W	1.5T	100 patients had profound neurological deficits and cord compression requiring surgical decompression	100% (100/100)
Como et al., 2012	SCIWORA	*n* = 24	Mean: 60.5, range: 34–83	Cervical	T1W, T2W	1.5T	13 patients required operative decompression	54% (13/24)
Boese et al., 2016	SCIWORA	*n* = 23	Mean: 53.7, range: 22–80	Cervical	T1W, T2W	1.5T	Only patients with both cord compression and intramedullary edema (classified as Type IIc) were considered for surgery, 8/15 of these underwent surgery	35% (8/23)
Maung et al., 2017	SCIWORA	*n* = 123	NS	Cervical	NS	NS	6 patients had MRI findings that led to surgical treatment (ligamentous injury, epidural hematoma)	5% (6/123)
Bao et al., 2020	SCIWORA	*n* = 16	Mean: 51.1, range: 30–73	Cervical	T1W, T2W	3.0T	10 patients received surgical treatment based upon neutral MRI results (cord compression, disc injury) and another 2 patients had surgery based on kinetic MRI showing instability	75% (12/16)
Huang et al., 2020 *	SCI, SCIWORA	SCIWORA: *n* = 42, SCI: *n* = 12	NS	Cervical	NS	3T, 1.5T	10 patients had MRI findings that led to surgical treatment (cord compression, ligamentous injury, disc herniation)	19% (10/54)
Meta-analysis ***	SCI, SCIWORA	*n* = 611		Mixed			Any finding leading to surgery	36% (223/611), I^2^ = 0.96, *p* < 0.001
**Surgical Approach**								
Kalfas et al., 1988	SCI	*n* = 62	NS	Cervical (*n* = 40), Thoracic (*n* = 17), Lumbar (*n* = 5)	T1W, T2W	0.5T	2 patients had cord compression due to acute disc herniation leading to anterior surgery	3% (2/62)
Mirvis et al., 1988	SCI	*n* = 21	Mean: 42.5, range: 17–66	Cervical	T1W, T2W	1.5T	3 patients with disc herniation were managed with anterior decompression	14% (3/21)
Doran et al., 1993	SCI	*n* = 12 (subgroup)	Mean: 34.1, range: 18–59	Cervical	NS	NS	10 patients with frank disc herniation and severe cord compression were managed with anterior cervical discectomy	83% (10/12)
Selden et al., 1999 **	SCI	*n* = 55	Mean: 29.2, range: 2–92	Cervical	T1W, T2W	1.5T	Among 18 patients with bilateral dislocated facets, acute disc herniation in 10/18 led to anterior surgery	18% (10/55)
Ghanta et al., 2002 *	SCIWORA	*n* = 13 (subgroup)	Mean: 28.5, range: 0.4–78	Cervical	NS	NS	1 patient with disc herniation was managed with anterior decompression	8% (1/13)
Tewari et al., 2004 **	SCIWORA	*n* = 40	Mean: 42.1, range: 16–70	Cervical	T1W, T2W	NS	3 patients with disc herniation were managed with anterior decompression	8% (3/40)
Sharma et al., 2009	SCIWORA	*n* = 12	Mean: 38.66, range: 22–58	Cervical	T1W, T2W	NS	2 patients had disc prolapse and underwent surgery due to this finding	17% (2/12)
Aarabi et al., 2011	SCIWORA, CCS	*n* = 211	Mean: 58.3, range: 32–87	Cervical	T2W, STIR	NS	Among 42 patients that required surgery, anterior approach was chosen in 28 due to anterior compression limited to 1–3 segments and/or kyphosis, while posterior was chosen in the remaining 14	20% (42/211)
Cheng et al., 2013	SCIWORA	*n* = 70	Mean: 57.7, range: 36–79	Cervical	T1W, T2W	NS	Among 70 patients treated surgically, MRI findings dictated surgical approach: 45 underwent anterior surgery due to anterior cord compression (disc, osteophytes, or OPLL); the remaining 25 underwent posterior procedures	100% (70/70)
Meta-analysis *	SCI, SCIWORA	*n* = 500		Mixed			Any finding leading to difference in surgical approach	29% (143/500), I^2^ = 0.97, *p* < 0.001
**When to Operate**								
Selden et al., 1999 **	SCI	*n* = 55	Mean: 29.2, range: 2–92	Cervical	T1W, T2W	1.5T	27 patients had cord compression leading to emergent surgery	49% (27/55)
Papadopoulos et al., 2002 **	SCI	*n* = 66	Mean: 32, range: 2–92	Cervical	T1W, T2W	1.5T	34 patients had cord compression leading to emergent surgery No cord compression in 32 patients after traction, allowing delayed surgery in 22 Total	51% (34/66) 33% (22/66) 85% (56/66)
Darsaut et al., 2006	SCI, fracture-dislocation	*n* = 17	Mean: 40.2; range: 19–78	Sub-axial (C3-T1)	T2W, T1W	1.5T	Among 11 patients with cord compression pre-reduction, MRI showed decompression in 9/11, leading to delayed surgery	53% (9/17)
Meta-analysis ***	SCI, SCIWORA	*n* = 83		Mixed			Any finding leading to difference in surgical timing	78% (65/83), I^2^ = 0.84, *p* = 0.01
**Need for Instrumentation**								
Krappinger et al., 2019 **	SCIWORA, CCS, hyperextension injury	*n* = 23	Mean: 62.7, range: 38–87	Cervical	T1W, T2W, STIR	1.5T	Findings of cord edema in all 23 patients and ligamentous injury (suggesting segmental instability) in 19 patients (including instability at a different level in several patients) led to decompression and instrumented fusion	100% (23/23)
**Which Levels or How Many Levels to Decompress**								
Krappinger et al., 2019 **	SCIWORA, CCS, hyperextension injury	*n* = 23	Mean: 62.7, range: 38–87	Cervical	T1W, T2W, STIR	1.5T	Findings of cord edema in all 23 patients and ligamentous injury (suggesting segmental instability) in 19 patients (including instability at a different level in several patients) led to decompression and instrumented fusion	100% (23/23)
**Need for Re-operation After Surgery**								
Aarabi et al., 2011 **	SCIWORA, CCS	*n* = 211	Mean: 58.3, range: 32–87	Cervical	T2W, STIR	NS	Among 28 patients that underwent anterior surgery, post-operative MRI found ongoing cord compression in 11, leading to additional posterior surgery	5% (11/211)
Aarabi et. al., 2019 **	SCI	*n* = 184	Mean: 43.5	Cervical	T1W, STIR	NS	Ongoing cord compression after surgery (inadequate decompression), but rates of re-operation were not reported	34% (63/184)

* Studies that include pediatric patients (<16) or unspecified age range. ** Studies with overlapping cohorts. *** Selden et al., 1999 was excluded from meta-analysis due to overlapping cohort with Papadopoulos et al., 2002.

**Table 6:** Key Question 4: When should MRI be performed in acute spinal cord injury?

Citation	Disease State	Sample Size	Age (Years)	SCI Level	Sequence	Field Strength	Evidence Regarding Timing of MRI
**Performance of MRI on Initial Assessment (Prior to Intervention)?**							
Kalfas et al., 1988	SCI	*n* = 62	NS	Cervical (*n* = 40), Thoracic (*n* = 17), Lumbar (*n* = 5)	T1W, T2W	0.5T	Useful to detect disc herniation, cord compression (if to operate, surgical approach)
Doran et al., 1993	SCI, facet dislocation	*n* = 12	Mean: 34.1, range: 18–59	Cervical	NS	NS	Useful to detect disc herniation, cord compression (if to operate, surgical approach)
Gupta et al., 1999	SCIWORA	*n* = 15	Range: 20–60	Cervical	NS	NS	Useful to detect disc herniation, cord compression (if to operate, surgical approach)
Selden et al., 1999 **	SCI	*n* = 55	Mean: 29.2, range: 2–92	Cervical	T1W, T2W	1.5T	Useful to detect disc herniation, cord compression (if to operate vs. closed reduction, surgical approach, timing of surgery)
Vaccaro et al., 1999	SCI, facet dislocation	*n* = 11	Mean: 46, range: 17–84	Cervical	T1W, T2W	1.5T	Unclear if pre-reduction MRI has utility: 2 patients had disc herniations prior to closed reduction but did not deteriorate after reduction
Papadopoulos et al., 2002 **	SCI	*n* = 66	Mean: 32, range: 2–92	Cervical	T1W, T2W	1.5T	Useful to detect cord compression (if to operate, timing of surgery)
Darsaut et al., 2006	SCI, fracture-dislocation	*n* = 17	Mean: 40.2, range: 19–78	Cervical	T1W, T2W	1.5T	Unclear if pre-reduction MRI has utility: 11 patients had cord compression prior to traction/reduction but did not deteriorate after reduction
Sharma et al., 2009	SCIWORA	*n* = 12	Mean: 38.66, range: 22–58	Cervical	T1W, T2W	NS	Useful to detect disc herniation, cord compression (if to operate, surgical approach)
Aarabi et al., 2011	SCIWORA, CCS	*n* = 211	Mean: 58.3, range: 32–87	Cervical	T2W, STIR	NS	Useful to detect anterior cord compression (surgical approach)
Como et al., 2012	SCIWORA	*n* = 24	Mean: 60.5, range: 34–83	Cervical	T1W, T2W	1.5T	Useful to detect cord compression (if to operate)
Cheng et al., 2013	SCIWORA	*n* = 70	Mean: 57.7, range: 36–79	Cervical	T1W, T2W	NS	Useful to detect anterior cord compression (surgical approach)
Boese et al., 2016	SCIWORA	*n* = 23	Mean: 53.7, range: 22–80	Cervical	T1W, T2W	1.5T	Useful to detect cord compression and edema (if to operate), but authors state “our results cannot provide guidance on therapeutic management”
Maung et al., 2017	SCIWORA	*n* = 123	NS	Cervical	NS	NS	Useful to detect ligamentous injury, epidural hematoma (if to operate)
Bao et al., 2020	SCIWORA	*n* = 16	Mean: 51.1, range: 30–73	Cervical	T1W, T2W	3.0T	Useful to detect cord compression, disc injury, instability (if to operate)
Huang et al., 2020 *	SCI, SCIWORA	SCIWORA: *n* = 42, SCI: *n* = 12	NS	Cervical	NS	3T, 1.5T	Useful to detect cord compression, ligamentous injury, disc herniation (if to operate)
**Performance of MRI After Closed Reduction to Assess Decompression?**							
Selden et al., 1999 **	SCI	*n* = 55	Mean: 29.2, range: 2–92	Cervical	T1W, T2W	1.5T	Useful to detect post-reduction cord compression (if to operate)
Vaccaro et al., 1999	SCI, facet dislocation	*n* = 11	Mean: 46, range: 17–84	Cervical	T1W, T2W	1.5T	Unclear if post-reduction MRI has utility: 5 patients had disc herniations after closed reduction but did not deteriorate
Darsaut et al., 2006	SCI, fracture-dislocation	*n* = 17	Mean: 40.2, range: 19–78	Cervical	T1W, T2W	1.5T	Useful to detect post-reduction cord compression (if to operate)
**Performance of MRI After Surgery to Assess Decompression?**							
Aarabi et al., 2011 **	SCIWORA, CCS	*n* = 211	Mean: 58.3, range: 32–87	Cervical	T2W, STIR	NS	Useful to detect post-operative cord compression (if to re-operate)
Aarabi et al., 2019 **	SCI	*n* = 184	Mean: 43.5	Cervical	T1W, STIR	NS	Useful to detect post-operative cord compression (if to re-operate), but rates of re-operation were not reported
**Performance of MRI within a Specific Time Period (e.g., 24 h)**							
Aarabi et al., 2019 *	SCI	*n* = 184	Mean: 43.5	Cervical	T1W, STIR	NS	Time interval to pre-operative MRI did not differ between successfully (8.3 +/− 7.7 h) and unsuccessfully (8.6 +/− 8.7 h) decompressed patients

* Studies that include pediatric patients (<16) or unspecified age range. ** Studies with overlapping cohorts.

**Table 7:** Key Question 5: What is the frequency of adverse events when performing MRI in acute SCI patients?

Citation	Disease	Sample Size	Age (Years)	SCI Level	Sequence	Field Strength	Activity/Imaging	Adverse Event	Outcome
Kalfas et al., 1988 *	SCI	*n* = 62	NS	Cervical (*n* = 40) Thoracic (*n* = 17) Lumbar (*n* = 5)	T1W, T2W	0.5T	MRI within first 36 h of injury	Any adverse event	0% (0/62)
Selden et al., 1999 **	SCI	*n* = 18	Mean: 29.2, range: 2–92	Cervical	T1W, T2W	1.5T	MRI within first 21 h of injury	Any adverse event	0% (0/55)
Papadopoulos et al., 2002 **	SCI	*n* = 66	Mean: 32, range: 2–92	Cervical	T1W, T2W	1.5T	Emergent MRI (average: 4.1 h)	Neurological deterioration	0% (0/66)
Darsaut et al., 2006	SCI, fracture-dislocation	*n* = 17	Mean: 40.2, range: 19–78	Cervical	T1W, T2W	1.5T	MRI during closed reduction	Permanent neurological deterioration during reduction/MRI Burning sensation at pin sites	0% (0/12) 0% (0/12)
Bao et al., 2020	SCIWORA	*n* = 16	Mean: 51.1, range: 30–73	Cervical	T1W, T2W	3.0T	Neutral, flexion, and extension MRI Neutral and flexion MRI	Neurological deterioration Neurological deterioration	0% (0/14) 0% (0/2)
Meta-analysis ***	SCI	*n* = 156	NS	Mixed	NS	NS		Any adverse event	0% (0/156), I^2^ = 0, *p* = 1

* Studies that include pediatric patients (<16) or unspecified age range. ** Studies with overlapping cohorts. *** Selden et al., 1999 was excluded from meta-analysis due to overlapping cohort with Papadopoulos et al., 2002.

**Table 8:** Key Question 6: How does obtaining an MRI (compared with not obtaining MRI) affect neurological, functional, and health-related quality of life outcomes? (Differences in outcome between patients receiving MRI and those not receiving MRI.).

Citation	Disease State	Sample Size	Age (Years)	SCI Level	Sequence	Field Strength	Outcome	Imaging/Treatment Group	Result	
**Improvement in Frankel Grade from Admission**										
Papadopoulos et al., 2002 **	SCI	*n* = 91	Mean: 32, range: 2–92	Cervical	T1W, T2W	1.5T	Any Frankel Grade improvement Grade A/B improvement to D/E	MRI-protocol Reference group MRI-protocol Reference group	50% (30/66) 24% (6/25) 16% (8/50) 0% (0/20)	*p* < 0.006 *p* = 0.09 ***
**Length of Stay**										
Papadopoulos et al., 2002 **	SCI	*n* = 91	Mean: 32, range: 2–92	Cervical	T1W, T2W	1.5T	ICU stay General care duration Rehabilitation duration Total length of stay	MRI-protocol Reference group MRI-protocol Reference group MRI-protocol Reference group MRI-protocol Reference group	9.9 ± 1.7 days 23.8 ± 3.7 days 8.4 ± 1.7 days 9.3 ± 3.0 days 58.1 ± 5.6 days 66.0 ± 10.7 days 71.4 ± 5.9 days 99.9 ± 13.1 days	*p* < 0.001 *p* = 0.31 *p* = 0.47 *p* = 0.02

** This study assigned patients non-randomly to either (1) an MRI-based protocol that included urgent imaging and treatment, or (2) a reference group that did not receive MRI or emergent surgical treatment. This study was deemed to have a high risk of bias, primarily due to selection. *** *p* value calculated using Fisher exact test, not reported by authors.

**Table 9:** Risk of bias assessment. Studies arranged alphabetically by the last name of the first author. Risk of bias assessment was performed according to the National Institutes of Health (NIH) Quality Assessment Tool. Studies appraised as good had minimally low risk of bias, studies appraised as fair had moderately low risk of bias, and those appraised as poor had high risk of bias.

	Study	Year	Study Design	Risk of Bias
1	Aarabi et al.	2011	Retrospective case series	Minimally low
2	Aarabi et al.	2019	Retrospective case series	Minimally low
3	Asan et al.	2018	Prospective case series	Moderately low
4	Bao et al.	2020	Retrospective case series	Moderately low
5	Boese et al.	2016	Retrospective case series	Moderately low
6	Cheng et al.	2012	Retrospective case series	Moderately low
7	Como et al.	2012	Retrospective case series	Moderately low
8	Darsaut et al.	2006	Prospective case series	Moderately low
9	Doran et al.	1993	Retrospective case series	High
10	D’Souza et al.	2017	Retrospective case control study	Moderately low
11	Fehlings et al.	1999	Retrospective case series	Moderately low
12	Ghanta et al.	2002	Retrospective case series	Moderately low
13	Gupta et al.	1999	Retrospective case series	Moderately low
14	Hendey et al.	2002	Retrospective case series	Moderately low
15	Henninger et al.	2020	Retrospective case series	Moderately low
16	Huang et al.	2020	Retrospective case series	Minimally low
17	Kalfas et al.	1988	Retrospective case series	Moderately low
18	Koyanagi et al.	2003	Retrospective case series	Moderately low
19	Krappinger et al.	2019	Retrospective case series	Minimally low
20	Liu et al.	2015	Retrospective case series	Minimally low
21	Machino et al.	2019	Retrospective case series	Minimally low
22	Maeda et al.	2012	Retrospective case series	Minimally low
23	Mahmood et al.	2010	Retrospective case control study	Moderately low
24	Maung et al.	2016	Retrospective case series	Moderately low
25	Mirvis et al.	1988	Retrospective case series	Moderately low
26	Papadopoulos et al.	2002	Prospective cohort study	High
27	Selden et al.	1999	Retrospective case series	Minimally low
28	Sharma et al.	2009	Retrospective case series	Minimally low
29	Song et al.	2008	Retrospective case series	Minimally low
30	Tewari et al.	2005	Retrospective case series	Minimally low
31	Vaccaro et al.	1999	Retrospective case series	Minimally low
32	Zhu et al.	2019	Retrospective case series	Minimally low

## Data Availability

All data supporting reported results can be found within the manuscript.

## Appendix A

**Pubmed (MEDLINE) Search Strategy**

(((magnetic resonance imaging) OR (MRI)) AND (((((((SCI) OR (spinal cord injury)) OR (spinal trauma)) OR (spine fracture)) OR (spine trauma)) OR (cervical fracture)) OR (cervical trauma))) AND ((((((((outcome) OR (recovery)) OR (management)) OR (decision-making)) OR (decision)) OR (surgery)) OR (surgical)) OR (treatment))

**Embase Ovid Search Strategy**

(magnetic AND resonance AND imaging OR mri) AND (((((((sci OR spinal) AND cord AND injury OR spinal) AND trauma OR spine) AND fracture OR spine) AND trauma OR cervical) AND fracture OR cervical) AND trauma) AND ((outcome OR recovery OR management OR decision) AND making OR decision OR surgery OR surgical OR treatment)

**CENTRAL Search Strategy**

[Magnetic Resonance Imaging] explode all trees OR MRI AND [Spinal Cord Injuries] explode all trees OR spinal cord injury OR SCI OR spinal trauma OR spine trauma OR spine fracture OR cervical fracture OR cervical trauma AND [Outcome Assessment, Health Care] explode all trees OR outcome OR recovery OR management OR MeSH descriptor: [Clinical Decision-Making] explode all trees OR decision-making OR decision OR MeSH descriptor: [General Surgery] explode all trees OR surgery OR surgical OR treatment OR MeSH descriptor: [Therapeutics] explode all trees.
